# The post-emergence period for denning Polar Bears: phenology and influence on cub survival

**DOI:** 10.1093/jmammal/gyae010

**Published:** 2024-03-02

**Authors:** Erik M Andersen, Ryan R Wilson, Karyn D Rode, George M Durner, Todd C Atwood, David D Gustine

**Affiliations:** U.S. Fish and Wildlife Service, Marine Mammals Management, 1011 E. Tudor Road, Anchorage, AK 99502, United States; U.S. Fish and Wildlife Service, Marine Mammals Management, 1011 E. Tudor Road, Anchorage, AK 99502, United States; U.S. Geological Survey, Alaska Science Center, 4210 University Drive, Anchorage, AK 99508, United States; U.S. Geological Survey, Alaska Science Center, 4210 University Drive, Anchorage, AK 99508, United States; U.S. Geological Survey, Alaska Science Center, 4210 University Drive, Anchorage, AK 99508, United States; U.S. Fish and Wildlife Service, Marine Mammals Management, 1011 E. Tudor Road, Anchorage, AK 99502, United States

**Keywords:** Alaska, behavior, den abandonment, human disturbance, maternity den, phenology, post-denning, sea ice, *Ursus maritimus*

## Abstract

Among polar bears (*Ursus maritimus*), only parturient females den for extended periods, emerging from maternal dens in spring after having substantially depleted their energy reserves during a fast that can exceed 8 months. Although den emergence coincides with a period of increasing prey availability, polar bears typically do not depart immediately to hunt, but instead remain at the den for up to a month. This delay suggests that there are likely adaptive advantages to remaining at the den between emergence and departure, but the influence of the timing and duration of this post-emergence period on cub survival has not been evaluated previously. We used temperature and location data from 70 denning bears collared within the Southern Beaufort Sea and Chukchi Sea subpopulations to estimate the phenology of the post-emergence period. We evaluated the influence of various spatial and temporal features on duration of the post-emergence period and evaluated the potential influence of post-emergence duration on litter survival early in the spring following denning. For dens that likely contained viable cubs at emergence (*n* = 56), mean den emergence occurred on 16 March (SE = 1.4 days) and mean departure on 24 March (SE = 1.6 days), with dates typically occurring later in the Chukchi Sea relative to Southern Beaufort Sea and on land relative to sea ice. Mean duration of the post-emergence period was 7.9 days (SE = 1.4) for bears that were observed with cubs later in the spring, which was over 4 times longer than duration of those observed without cubs (1.9 days). Litter survival in the spring following denning (*n* = 31 dens) increased from 0.5 to 0.9 when duration of the post-emergence period increased by ~4 days and other variables were held at mean values. Our limited sample size and inability to verify cub presence at emergence suggests that future research is merited to improve our understanding of this relationship. Nonetheless, our results highlight the importance of the post-emergence period in contributing to reproductive success and can assist managers in developing conservation and mitigation strategies in denning areas, which will be increasingly important as human activities expand in the Arctic.

## Teaser

Polar bears observed with cubs in the spring following denning stayed at the den site 4 times longer than those observed without cubs, and cub survival during that period increased from 0.5 to 0.9 when time spent at the den increased from 0 to 4 days.

For black (*Ursus americanus*) and brown bears (*U. arctos*) that inhabit northern regions where food is largely unavailable during winter months, denning is a strategy utilized by all ages and sexes to conserve energy (Nelson et al. 1983; González-Bernardo et al. 2020). For polar bears (*U. maritimus*), however, prey are available throughout winter. Consequently, only parturient polar bears den for extended periods during the winter, which suggests that denning strategies should function primarily to maximize reproductive success (Rode et al. 2018b). Polar bears excavate maternal dens in snowdrifts on land or sea ice in late fall or early winter and give birth to cubs in December or January (Derocher et al. 1992; Amstrup and Gardner 1994; Messier et al. 1994). Dens provide the altricial neonates with the warmth and protection they require until they are capable of surviving surface conditions upon emergence (>2 months postpartum; Blix and Lentfer 1979; Ramsay and Dunbrack 1986; Amstrup 1993).

Polar bears typically emerge from dens in March and April (Rode et al. 2018b), a time of increasing availability of their primary prey, sea ice-dependent seals, particularly ringed seals (*Pusa hispida*) and bearded seals (*Erignathus barbatus*). Both seal species give birth to young during March and April, either in subnivean lairs (*P. hispida*) or on the exposed surface of ice flows (*E. barbatus*), and the adults and juveniles of both seals must haul out onto ice to molt during April (Smith 1980; Cameron et al. 2010; Kelly et al. 2010). Although adult female polar bears do not eat during denning and can lose up to 43% of their body mass during a fast that can surpass 8 months (Atkinson and Ramsay 1995), family groups typically do not depart the den site immediately after emergence but instead can remain for up to a month before departing to hunt (Jonkel et al. 1972; Hansson and Thomassen 1983; Smith et al. 2013). This delay in the onset of hunting suggests that there may be adaptive advantages to remaining at the den site after emergence. During the period between den emergence and departure (henceforth 'post-emergence'), family groups spend time inside and outside the den, likely acclimating physiologically to external conditions. Cubs are active (e.g. playing, walking, digging) the majority of the time they spend outside the den (Smith et al. 2007, 2013), and activity increases with time since emergence (Hansson and Thomassen 1983), which suggests that the post-emergence period may be an important time for cubs to build the strength necessary for traveling across sea ice and traversing open leads (Hansson and Thomassen 1983; Messier et al. 1994).

Climate change has resulted in spatiotemporal reductions in the quantity and quality of sea ice habitat for polar bears (Stern and Laidre 2016; Durner et al. 2019), which in turn has led to increases in summer land use in the Southern Beaufort Sea (hereafter, SB) and Chukchi Sea (hereafter, CS) subpopulations (Rode et al. 2015; Atwood et al. 2016), increases in denning on land in the SB (Fischbach et al. 2007; Olson et al. 2017), and since 2000 a westward shift of land denning on the Alaska Beaufort Sea coastal plain (Patil et al. 2022). This increase and westward shift in land denning has coincided with an escalation of anthropogenic activity in the Alaska Arctic (Wilson et al. 2014; Rode et al. 2018a), increasing the potential for disturbance to denning polar bears (Atwood et al. 2017; Wilson and Durner 2020). Disturbance events have been linked to den abandonment (i.e. den departure earlier than would have occurred under undisturbed conditions) for a variety of bear species, including polar bears (Amstrup 1993; Linnell et al. 2000; Woodruff et al. 2022). Survival of polar bear cubs increases with body mass and is lowest within the first few months after den departure (Derocher and Stirling 1996). Consequently, den abandonment can expose cubs to various threats or stressors before they attain the size, strength, and level of acclimation to external conditions that they would have attained if undisturbed. This can reduce their ability to traverse sea ice topography (Ovsyanikov 1998) and could increase the risk of mortality from hypothermia, starvation, or predation (Blix and Lentfer 1979; Derocher and Stirling 1996; Amstrup et al. 2006). Denning bears may be especially susceptible to disturbance during the post-emergence period, when opened dens are increasingly vulnerable to external stimuli (Owen et al. 2021) and bears are regularly present outside the den (Smith et al. 2007).

Despite the potential importance of the post-emergence denning period to reproductive success and increased potential for detrimental impacts from climate change and disturbance, the influence of the timing and duration of the post-emergence period on cub survival has not been evaluated. Much of what is known about the phenology of this denning stage comes from studies where polar bear dens were observed from blinds or by video cameras, where the observation process inherently involves potential disturbance to dens (Smith et al. 2013; Larson et al. 2020). Additionally, observational studies during the post-emergence period generally focused on areas where dens occurred at high densities (e.g. Hansson and Thomassen 1983; Ovsyanikov 1998) or near industrial activities centered in Prudhoe Bay, Alaska (e.g. Smith et al. 2007, 2013; Robinson 2014), in part because of the logistical difficulties inherent to locating and monitoring dens on sea ice (which moves on currents and with wind) or in remote areas where denning occurs at low densities (Messier et al. 1994).

Temperature data recorded by thermistors integrated into radio collars have been used to successfully identify dates of den entrance and emergence by polar bears (Olson et al. 2017; Rode et al. 2018b). This approach has several advantages over other methods, including the ability to monitor phenology at dens not exposed to human activity and those that occur on sea ice, where sea ice drift confounds the identification of denning events from location data. With this method, Olson et al. (2017) and Rode et al. (2018b) identified denning as the period in a time series of temperature data when mean daily temperatures recorded on collars exceeded the expected variation in collar temperature from bears known to have not denned that year. Their temperature-based approach correctly classified denning and nondenning behavior in 95% of bears (*n* = 73) when compared to direct observations (Olson et al. 2017). Estimates of entrance into dens on land derived by this method did not differ from those estimated with location data; estimates of emergence dates, however, were 2.8 days earlier than those estimated with location data, but the authors note that location-based estimates represent departure from the den site, whereas temperature-based estimates represent the opening of a den (Rode et al. 2018b).

Here, we expanded the approach developed by Olson et al. (2017) to identify the phenology and duration of the post-emergence period for polar bears collared within the boundaries of SB and CS subpopulations (Obbard et al. 2010). We estimated dates of den emergence and departure using temperature thresholds specific to each den to account for variation in den temperatures and thermistor accuracy across dens, and we compared estimates of den departure derived from temperature data to those derived from location data. Additionally, we evaluated the influence of various spatial and temporal features on the duration of the post-emergence period and estimated the potential influence of post-emergence duration on litter survival in the spring following denning.

## Materials and methods

We obtained collar temperature and location data from adult (≥4 years old) female polar bears equipped with satellite radio collars (Telonics, Inc., Mesa, Arizona) that were captured within the boundaries of SB (1985 to 2016) or CS subpopulations (2008 to 2017). Collars were deployed on bears located from a helicopter and immobilized with standard techniques (Stirling et al. 1989) near the northern and northwestern coasts of mainland Alaska between mid-March and early May (details on capture and immobilization are described further in Rode et al. 2015 and Atwood et al. 2016). Observations of previously collared bears occurred during these capture efforts, and the presence and age of dependent young were recorded. Radio collars recorded Argos (CLS 2016) or GPS location data at intervals of 1 h to 5 days and temperature data at intervals of 5 min to 12 h. For analyses, we used data spanning January through May, considered each bear-year to be an independent sample, and excluded data from years when bears did not den. Because our method for identifying den emergence (described below) was based on the mean January collar temperature, we considered only those bear-years with ≥5 temperature records for January (*n* = 105). We considered temperature records >40 °C to be implausible and excluded them from analyses (Tchernova 2010).

### Identifying den emergence and departure with temperature data

**Fig. 1.**
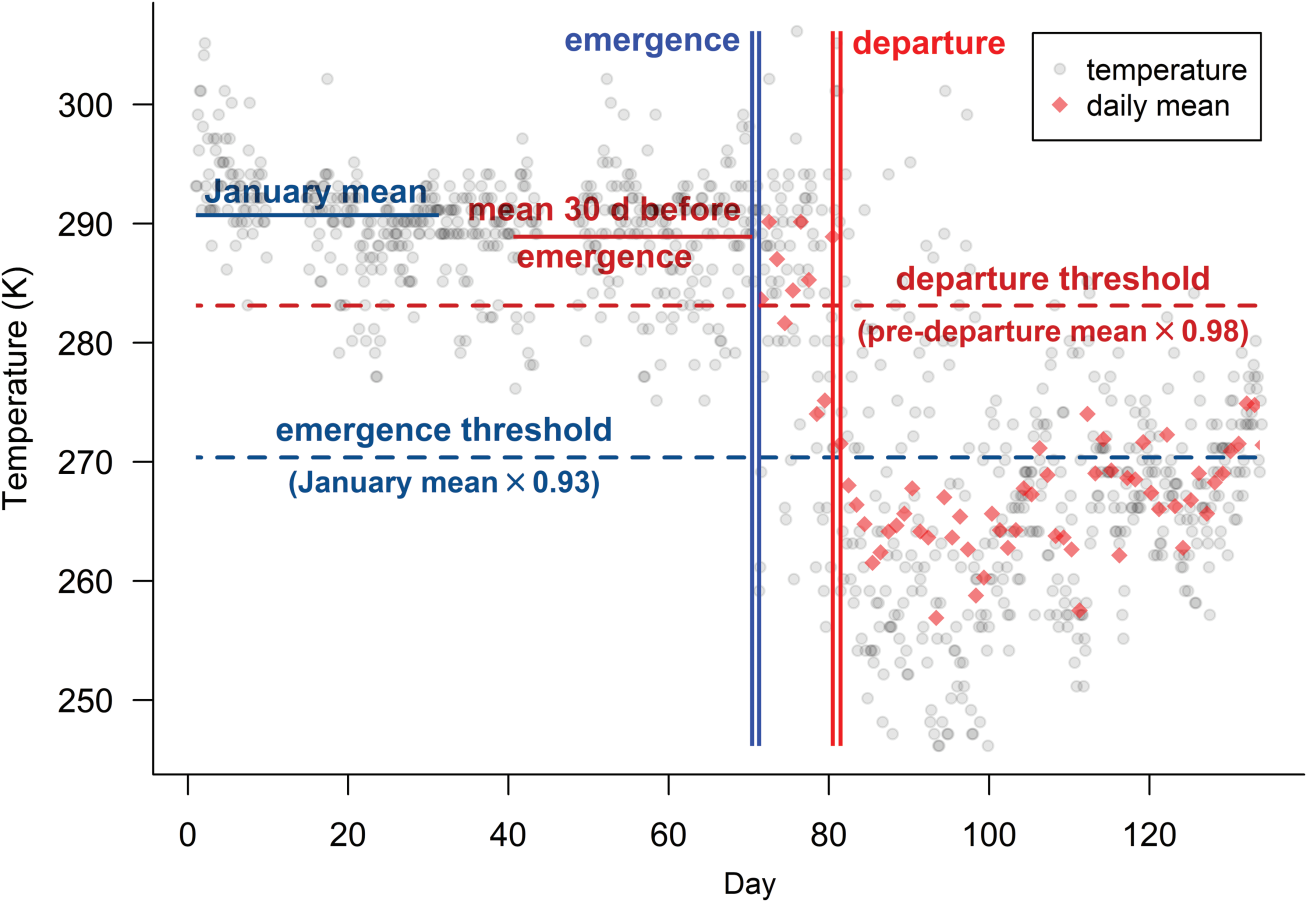
Example of temperature records (gray circles) recorded at a 3-h interval and daily temperature means post-emergence (red diamonds) recorded by a collar thermistor on a denning polar bear. Maximum emergence date ($E_{max}$, right vertical blue line) is estimated as the first occurrence of temperatures below the emergence threshold, which is defined by multiplying the mean January temperature recorded by the sensor by 0.93. The estimated minimum emergence date ($E_{min}$, left vertical blue line) is the record immediately before the maximum estimate. Similarly, the maximum departure date ($D_{max}$, right vertical red line) is estimated as the cessation of mean daily temperatures above the departure threshold, which is defined by multiplying the mean temperature recorded at the den 30 days prior to emergence by 0.98. The estimated minimum departure date ($D_{min}$, left vertical red line) is the first day with temperature records before the maximum estimate.


For each den, we estimated dates of den emergence and departure as the earliest occurrence of collar temperature records in the time series that surpassed thresholds defined by period-specific temperatures at that den (Fig. 1). In our study area, ambient temperatures during denning (e.g. mean January temperature is approximately −26 °C in Deadhorse, Alaska and −24 °C in Wrangel Island, Russia; Lozhkin et al. 2001; NOAA 2021) are markedly lower than those that typically occur in unopened dens (e.g. the temperature inside a sealed den was 21 °C warmer than ambient temperature; Harington 1968). Similar to prior investigations, we anticipated that den emergence would be characterized by a marked drop in temperature when the den was opened and exposed to colder ambient air (Fischbach et al. 2007; Olson et al. 2017). Specifically, we estimated den emergence as the earliest date in the time series when

$$\begin{array}{l} t_{i}<{\bar{t}}_{Jan}\times 0.93 \end{array}$$

where $t_{i}$ is the collar temperature (Kelvin) recorded at time *i* and ${\bar{t}}_{Jan}$ is the mean January collar temperature recorded at the den (Fig. 1). We used the Kelvin scale, in which the zero point represents absolute zero, to avoid arbitrary temperature proportions and because the temperatures we considered typically spanned 0 in other common scales. The constant 0.93 was selected because it was the proportion required to reduce the mean January temperature of all bears known to have denned by 20 °K, the approximate difference between ambient and unopened den temperatures (Harington 1968; Olson et al. 2017). Because the opening of a den should result in consistently lower temperatures relative to those recorded before the den was opened, we required that a second record below the threshold occur within 7 days to ensure that estimates were not based on measurement errors—because we selected 7 days arbitrarily, we examined the sensitivity of emergence estimates to this choice by comparing estimates based on 7 days to those estimated with 3, 5, 9, and 11 days.

Because bears can spend >97% of their time in the den during the post-emergence period (Smith et al. 2013; Robinson 2014), we expected average temperatures during that time to be cooler than before emergence but characterized by large variation attributable to the recording of temperatures both inside and outside the opened den. When bears departed the den site, we anticipated that mean daily temperatures would decrease because bears would no longer be spending time inside dens that were warm relative to ambient conditions. Hence, we defined the date of den departure as the first day in the time series after emergence when the mean daily collar temperature decreased below a den-specific threshold and remained below that threshold for at least 7 days. This threshold temperature was defined as


$$\begin{array}{l} t_{d}<{\bar{t}}_{den}\times 0.98 \end{array}$$

where $t_{d}$ is the mean daily temperature on day *d* and ${\bar{t}}_{den}$ is the mean temperature recorded during the 30 days prior to emergence (Fig. 1). Visual inspection of data suggested that temperatures recorded throughout denning were relatively consistent at most dens, but for some, temperature increased or decreased systematically as denning progressed, possibly due to changes in the denning environment (e.g. changes in the depth of insulating snow). Consequently, temperatures recorded 30 days prior to emergence provided a more representative baseline of conditions prior to departure on which to base the threshold than those recorded over longer periods of time. The constant 0.98 is the proportion required to reduce the mean ${\bar{t}}_{den}$ of bears known to have produced cubs (from subsequent observations) by 1 SD; this value was selected because it maximized congruence with GPS location-based departure estimates (described below) during preliminary analyses. We evaluated the sensitivity of den departure estimates to the choice of requisite number of days (7) that temperatures remained below the threshold with the same approach we used for emergence estimates.

For both den emergence (*E*) and departure (*D*), estimates represent the latest date at which the event occurred (i.e. the event was estimated to have occurred by this date because temperatures had exceeded the threshold); however, it is possible that the event actually occurred between the estimated date and previous date with temperature data, a period that could span several days if data were recorded infrequently. To account for variability in recording intervals across sensors, we refer to the dates estimated by methods described above as the maximum date of the event ($E_{max}$ and $D_{max}$ for emergence and departure, respectively) and the first date with temperature data prior to the estimate as the minimum date of the event ($E_{min}$ and $D_{min}$). Accordingly, we calculated the minimum duration of the post-emergence period ($Dur_{min}$) as $D_{min}-E_{max}$ and maximum duration ($Dur_{max}$) as $\;D_{max}-E_{min}$; negative duration values, which result when the range of emergence and departure estimates overlap, were treated as zeros. Midpoint duration was calculated as $\frac{Dur_{min}+\;Dur_{max}}{2}$. For example, at a den where temperature data were recorded at a 4-day interval, if the first day in the time series with temperatures below the emergence threshold was 10 March (and assuming a second record occurred below the threshold within 7 days), then we assumed emergence occurred between 6 March ($E_{min}$) and 10 March ($E_{max}$) with a midpoint emergence estimate of 8 March. To ensure that marked gaps in time series data due to variation in recording intervals did not lead to erroneous inference about denning phenology, we considered only those dens that included temperature data on >25% of days during the period spanning 7 days prior to $E_{min}$ to 7 days after $D_{max}$ (*n* = 70; Table 1). Unless indicated otherwise, reported dates and durations represent the midpoint between minimum and maximum estimates.

**Table 1.** Analyses and corresponding data from dens (*n*) of polar bears collared in the SB and CS subpopulations.

Analysis	Data	*n*	Years
Summaries of denning phenology	Dens with temperature data on >25% of days spanning 7 days before emergence to 7 days after departure^a^	70	1986 to 2018
Comparison of emergence and departure dates between subpopulations and substrates (*t*-tests); influence of variables on post-emergence duration (GLM)	Dens assumed to have produced cubs (maximum estimate of emergence ≥27 February)	56	1987 to 2018
Comparison of departure estimates from location versus temperature data (*t*-test)	Dens on land with estimates of departure derived from both temperature and location data	44	1989 to 2018
Litter survival (logistic regression)	Denning bears observed in spring following denning	31	1987 to 2015

GLM = generalized linear model.

^a^All other analyses were conducted with a subset of these data.

We evaluated the accuracy of temperature-based estimates of departure from dens on land through comparison with estimates derived from location data; we did not consider bears that denned on sea ice because their movements are confounded by drifting sea ice. For dens on land with location data spanning the period of temperature-based estimates of den departure (*n* = 44; Table 1), we used GPS locations and Argos location classes L3, L2, and L1 to identify the date bears moved >2,500 m from the den. We used a threshold of 2,500 m to account for location errors associated with these Argos classes (Douglas et al. 2012) and because typical movements of bears prior to den departure do not exceed this distance (Uspenski and Kistchinski 1972; Smith et al. 2013). As with temperature estimates, we considered the date of first location beyond this threshold to represent the maximum departure estimate and the date of the previous record in the time series to represent the minimum departure estimate. We used a paired *t*-test to compare departure date midpoints estimated with temperature data versus those estimated with location data and calculated the percentage of temperature-based estimates that fell within the range of location-based estimates. If temperature-based estimates of departure for a den were outside of the range of location-based estimates or overlapped but were less precise (i.e. the span between minimum and maximum estimates was larger), we considered location-based estimates to be more accurate and used those dates for subsequent analyses.

### Statistical analyses

Our primary focus was to understand the phenology and ecological significance of the post-emergence period for bears that reproduced successfully (i.e. emerged from the den with cubs). Our data set, however, likely included short-duration denning events that were not true maternal dens (i.e. shelter dens; Ferguson et al. 2000) and maternal dens that were unsuccessful due to implantation failure, abortion, or neonatal mortality (Uspenski and Kistchinski 1972; Derocher et al. 1992). Most shelter denning occurs between mid-December and late January (Messier et al. 1994), suggesting that dens with later emergence dates likely represent maternal dens. Because parturition typically occurs between late November and early January (Messier et al. 1994; Van De Velde et al. 2003) and cubs lack the ability to thermoregulate and move efficiently outside of the den until they reach a minimum of 60 to 70 days of age (Blix and Lentfer 1979; Kenny and Bickel 2005), emergences in January and early February are less likely to represent successful denning than those that occur later. In our data set, the earliest emergence date for a den known to be productive—based on the observation of cubs with the mother during surveys the spring following denning—was between 25 ($E_{min}$) and 27 February ($E_{max}$). Accordingly, to reduce the potential for bias resulting from the inclusion of nonmaternal and unsuccessful dens, which we expect to be characterized by earlier emergences and shorter post-emergence durations than successful dens, we excluded bears with $E_{max}$ dates <27 February (*n* = 14 of 70 dens) from analyses. While it is possible that dens with emergence dates ≥27 February may not have produced cubs, we assume that most failed pregnancies would manifest earlier, and in the absence of viable cubs, bears would be unlikely to remain in the den beyond this point. Although this assumption is supported by 3 studies in northern Alaska that collectively monitored 25 dens on land throughout March, all of which contained viable cubs at emergence (Smith et al. 2007, 2013; Robinson 2014), we note that cub presence at emergence could not be verified for dens in our analysis.

For the 56 dens assumed to have produced cubs, we used *t*-tests to compare mean dates of emergence and departure between subpopulations and denning substrates (Table 1). Additionally, we evaluated the influence of spatial and temporal features on duration of the post-emergence period with a generalized linear model (Table 1). These features included subpopulation, den substrate (land or sea ice), year, and emergence date (midpoint Julian day); we also included a quadratic term for emergence date because visual inspection of the data suggested the potential for a nonlinear pattern with a peak in post-emergence duration associated with intermediate emergence dates. Model variables were not correlated strongly with each other (|*r*| < 0.5), and variance inflation factors were <1.4, suggesting an absence of collinearity (Midi et al. 2010). We included an interaction between emergence date and den substrate to determine whether the influence of emergence date varied between dens on land and sea ice. We also included interactions between subpopulation and emergence date and year to determine whether the influence of these variables on post-emergence duration varied between SB and CS subpopulations. We standardized all numeric variables to *Z*-scores (mean = 0, SD = 1) and specified a log link function and a Poisson error distribution in the model.

We tested for an influence of post-emergence duration on litter survival early in the spring following denning after accounting for other variables associated with cub survival (Rode et al. 2018b; Bromaghin et al. 2021). Specifically, for bears with estimates of post-emergence duration that were observed during capture efforts in the spring following den departure when the presence (*n* = 25) or absence (*n* = 6) of cubs was recorded (mean days after departure = 25.5, SE = 3.1, range = 0 to 74; Table 1), we fit 8 logistic regression models with litter presence/absence as the dependent variable and used Akaike information criterion adjusted for small sample sizes (AIC_c_) to select a model for inference. Each model included 2 independent variables that have been shown previously to affect litter survival (Rode et al. 2018b)—emergence date and denning substrate. Additionally, the candidate model set included each combination of 3 other variables: post-emergence duration; survival interval duration (i.e. the number of days between den departure and observation in the spring); and the winter (January to March) Arctic Oscillation Index (AOI; NOAA 2022), which is an index of sea level pressure anomalies north of 20°N that has a strong effect on spring ice conditions (Rigor et al. 2002) and is associated with polar bear recruitment (Rode et al. 2021) and rates of ringed seal predation by polar bears (Pilfold et al. 2015; Table 2). Model variables were not correlated strongly with each other (|*r*| < 0.5), and variance inflation factors were <2.1, suggesting an absence of collinearity (Midi et al. 2010). The number of days observations occurred after departure was similar for bears observed with (mean = 24.8) and without (28.2) cubs-of-the-year. Of the 31 bear observations in the analysis, 30 occurred in the SB and 1 occurred in the CS, where most denning occurs in Russia and post-denning bears rarely reach sampling areas near Alaska in the spring when surveys occur. We standardized all numeric values as described above. Unless indicated otherwise, values are reported ± 1 SE.

**Table 2.** Results of model selection based on Akaike Information Criteria (corrected for small sample sizes; AIC_c_) used to evaluate the potential influence of duration at the den site after emergence on survival of 31 polar bear litters in the spring following denning. Logistic regression models related the presence or absence of cubs when adult females were observed during surveys in the spring following departure from the den site to combinations of 5 explanatory variables: denning substrate (i.e. sea ice or land) and emergence date, which were included in all models; the number of days after departure that observation occurred (observation); winter AOI; and the duration of the post-emergence period (duration). Females that exited dens prior to 27 February were excluded from the analysis due to the expectation that they emerged from dens without cubs.

Model variables	∆AIC_c_	df	*w*_*i*_
Substrate + emergence + duration + observation	0.00	26	0.45
Substrate + emergence + duration	1.84	27	0.18
Substrate + emergence + duration + observation + AOI	1.88	25	0.18
Substrate + emergence + duration + AOI	2.79	26	0.11
Substrate + emergence	4.38	28	0.05
Substrate + emergence + AOI	6.77	27	0.02
Substrate + emergence + observation	7.18	27	0.01
Substrate + emergence + observation + AOI	9.86	26	0.00

## Results

For the 105 dens we considered, emergence and departure dates were estimated from temperature data for 87 (83%); failure to produce an estimate was primarily due to time series data ending prior to the cessation of denning (data available per USGS and USFWS 2023). Seventy dens (SB, *n* = 47; CS, *n* = 23) representing 66 unique individuals had temperature data on >25% of days during the period spanning 7 days prior to $E_{min}$ to 7 days after $D_{max}$ and consequently were included in phenology estimates (Table 3). For these dens, the mean number of temperatures recorded per day of data acquisition was 14.2 ± 3.7. Midpoint departure dates estimated with temperature data did not differ from those estimated with location data (paired *t*-test: *t*_43.0_ = 0.32, *P* = 0.75); 86% of temperature-based estimates were within 1 day of location-based estimates and 95% were within 3 days. When different, temperature-based estimates typically were earlier than location-based estimates (69% of those that differed; Supplementary Data SD1). Altering the number of days temperature was required to remain below the threshold to estimate denning events by 2 days (i.e. from 7 days to 5 and 9 days) did not change emergence or departure estimates for 88% and 83% of dens, respectively. Similarly, altering the value by 4 days (i.e. 3 and 11 days) did not change emergence (84%) or departure (60%) estimates for most dens.

**Table 3.** Estimated midpoint dates of den emergence (*E*_mid_), departure (*D*_mid_), and duration at the den site post-emergence (Duration_mid_) for dens in 4 groups: (1) all dens; (2) dens that likely contained viable cubs at emergence based on an estimated maximum emergence date ≥27 February; (3) dens that were known to be successful because the adult female was later observed with cubs in the spring following denning; and (4) dens from which the adult female was later observed without cubs. For the latter group, summaries are provided for dens with emergence dates < and ≥27 February.

		*E*_mid_		*D*_mid_		Duration_mid_		
Group	*n*	Mean	*SE*	Mean	*SE*	Mean	*SE*	Range
All dens	70	7 March	2.6	13 March	3.0	7.2	0.9	0 to 31.5
Cubs likely present	56	16 March	1.4	24 March	1.6	8.8	1.0	0 to 31.5
Observed with cubs	25	17 March	2.3	24 March	2.2	7.9	1.4	0.5 to 22.5
Observed without cubs	11	23 February	7.2	24 February	6.0	1.9	0.7	0 to 7.5
Emerged <27 February	5	2 February	7.3	2 February	7.2	0.8	0.4	0 to 2.5
Emerged ≥27 February	6	12 March	4.4	14 March	4.5	2.8	1.1	0.5 to 7.5

**Fig. 2.**
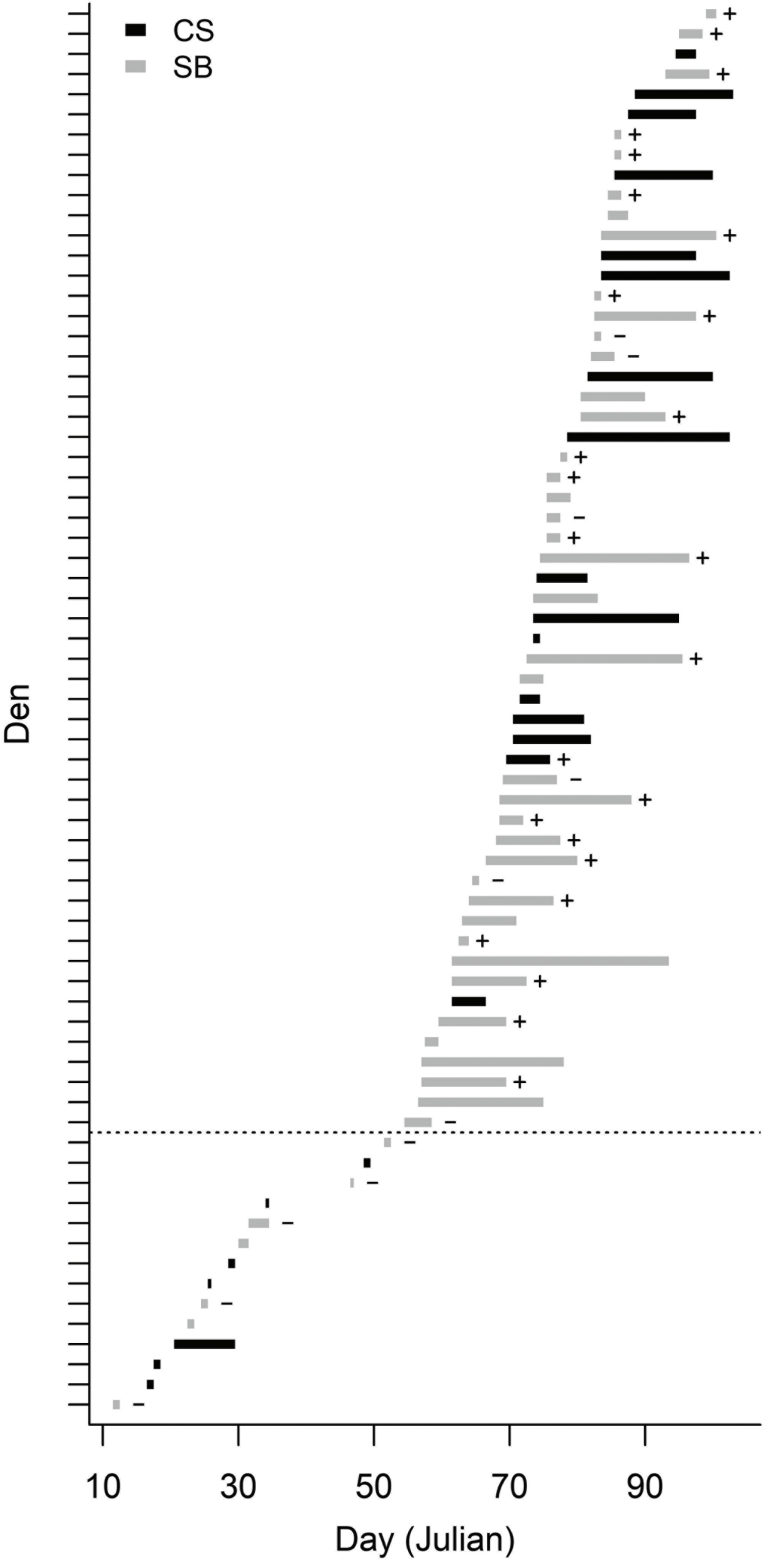
Estimated midpoint dates of den emergence (left side of bars) and departure (with 0.5 days added to improve visibility; right side of bars) and associated post-emergence duration for 70 polar bear dens in the SB and CS subpopulations. Denning bears observed with cubs in the spring following denning are denoted with a '+'; those observed without cubs are denoted with a '−'. Dens below the dotted line had maximum estimated emergence dates <27 February and were consequently considered not to contain viable cubs at emergence for purposes of analysis.

Denning phenology varied markedly across dens, with dates of den emergence ranging from 12 January to 9 April (all estimates represent midpoint dates unless noted otherwise; Fig. 2). Mean emergence was 17 March (± 2.3 days) for bears observed with cubs later in the spring (i.e. those for which successful reproduction was confirmed; *n* = 25) and 16 March (± 1.4 days) for dens assumed to have contained viable cubs at emergence (i.e. emergence dates ≥27 February; *n* = 56). Mean emergence was 23 February (± 7.2 days) for bears observed without cubs later in the spring (*n* = 11); of those, mean emergence from dens assumed to contain viable cubs at emergence was 12 March (± 4.4 days, *n* = 6; Table 3). Departure dates largely overlapped emergence dates, with dates of den departure ranging from 12 January to 13 April (Fig. 2). Mean departure was 24 March for bears observed with cubs later in the spring (± 2.2 days) and dens assumed to have contained viable cubs at emergence (± 1.6 days). Mean departure was 24 February (± 7.3 days) for bears observed without cubs later in the spring; of those, mean departure from dens assumed to contain viable cubs at emergence was 14 March (± 4.5 days; Table 3). Mean duration of the post-emergence period was 7.9 ± 1.4 days (range = 0.5 to 22.5 days) for bears observed with cubs later in the spring and 8.8 ± 1.0 day (range = 0 to 31.5 days) for dens assumed to have contained cubs at emergence. Mean duration of the post-emergence period was 1.9 ± 0.7 days (range = 0 to 7.5 days) for bears observed without cubs later in the spring; of those, mean duration at dens assumed to contain viable cubs at emergence was 2.8 days (± 1.1; Table 3).

For dens assumed to have contained cubs at emergence (*n* = 56), we found weak evidence for a difference in emergence dates between subpopulations (*t*_35.7_ = 1.64, *P* = 0.11), with mean emergence occurring on 14 March (± 1.8 days) for SB and 19 March (± 2.2 days) for CS subpopulations. Departure dates differed between subpopulations (*t*_25.5_ = 2.30, *P* = 0.03), with mean departure occurring on 22 March (± 1.8 days) for SB and 30 March (± 3.1 days) for CS subpopulations. Emergence dates did not differ between dens on sea ice or land (*t*_36.2_ = 1.17, *P* = 0.25), with mean emergence occurring on 13 March (± 2.3 days) for dens on sea ice and 19 March (± 1.8 days) for dens on land. We found weak evidence for a difference in departure dates (*t*_44.4_ = 1.59, *P* = 0.12), however, with mean departure occurring on 18 March (± 2.2 days) for dens on sea ice and 25 March (± 2.1 days) for dens on land.

**Fig. 3.**
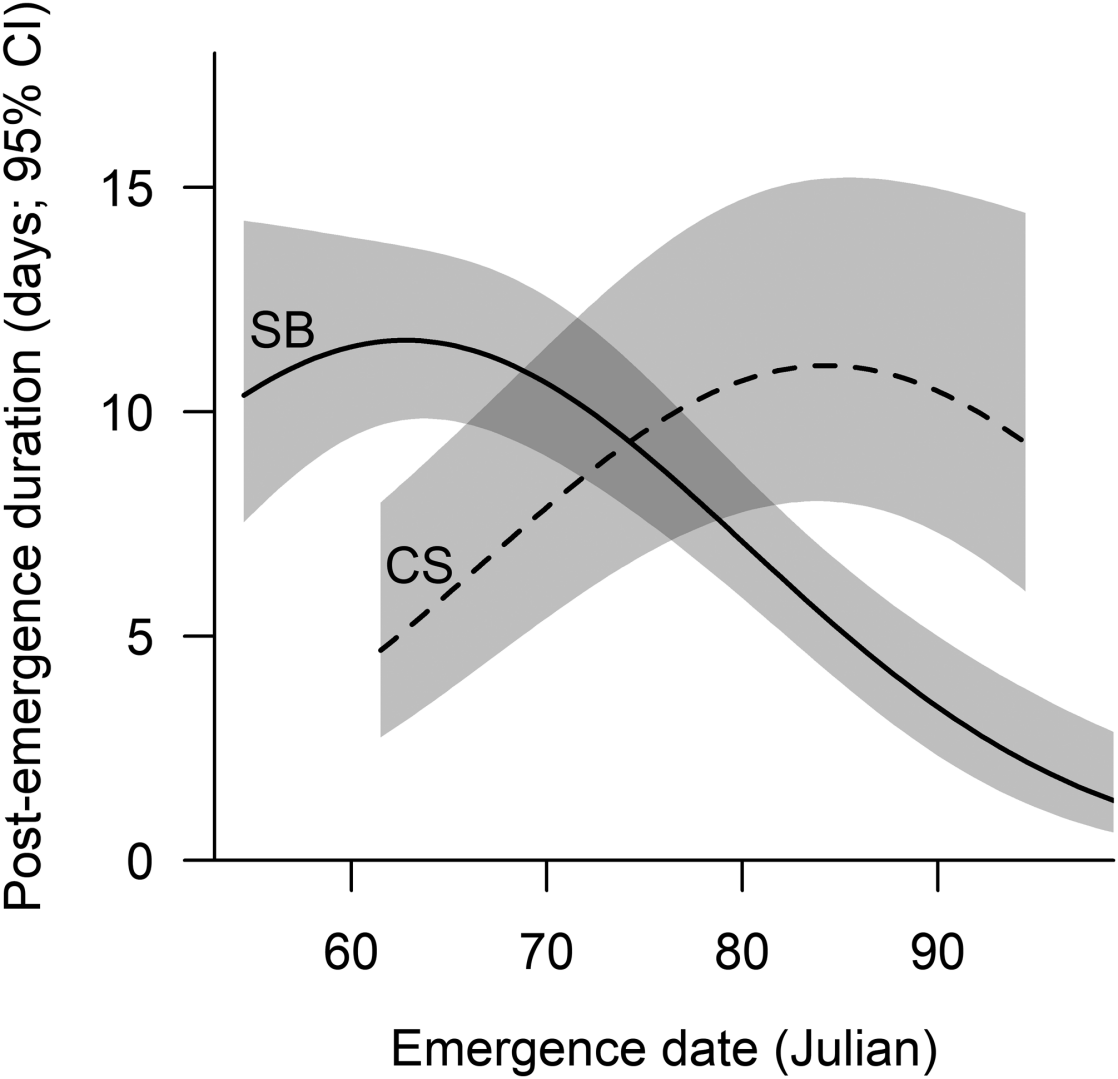
Expected influence of emergence date on the duration of the post-emergence period at 56 maternal dens of polar bears in the SB (solid line) and CS (dashed line) subpopulations. The reference level for den substrate was land and year was held constant at the mean value (2007).

Of features potentially associated with post-emergence duration at 56 dens assumed to have produced cubs, duration was influenced (*P* < 0.05) only by emergence date (emergence date^2^, *P* = 0.001), the effect of which differed between subpopulations (emergence date × subpopulation interaction, *P* < 0.001; Table 4). In the SB, predicted post-emergence duration decreased with increasing emergence date after a peak in early March (when other variables were held at mean values and “land” used as the reference substrate; Fig. 3). In the CS, however, post-emergence duration increased with increasing emergence date until late March when duration began to decrease (Fig. 3).

**Table 4.** Coefficient estimates (Est), standard errors (*SE*), test statistics (*Z*), and *P*-values (*P*) for intercept and covariate effects from models used to evaluate the influence of spatial and temporal features on the duration of the post-emergence period (PE duration; *n* = 56) and litter survival until the spring following den emergence (when bears were observed during surveys; *n* = 31) for polar bears in the SB and CS subpopulations. Reference levels for factors are in parentheses. Females that exited dens prior to 27 February were excluded from these analyses due to the likelihood that they emerged from dens without cubs.

Model	Covariate	Est	*SE*	*Z*	*P*
PE duration	Intercept	2.08	0.20	10.46	<0.001
	Emergence date	0.18	0.18	1.02	0.310
	Emergence date^2^	−0.19	0.06	−3.20	0.001
	Year	0.27	0.19	1.42	0.156
	Subpopulation (SB)	−0.03	0.18	−0.15	0.879
	Substrate (land)	0.16	0.13	1.28	0.202
	Subpopulation (SB) × emergence date	−0.77	0.15	−5.16	<0.001
	Subpopulation (SB) × year	−0.17	0.20	−0.85	0.394
	Substrate (land) × emergence date	0.17	0.14	1.17	0.242
Litter survival	Intercept	1.51	1.34	1.13	0.259
	Duration of survival interval	1.93	1.06	1.81	0.070
	Emergence date	2.29	1.11	2.06	0.039
	Substrate (land)	2.36	1.50	1.58	0.114
	PEduration	4.08	2.02	2.01	0.044

**Fig. 4.**
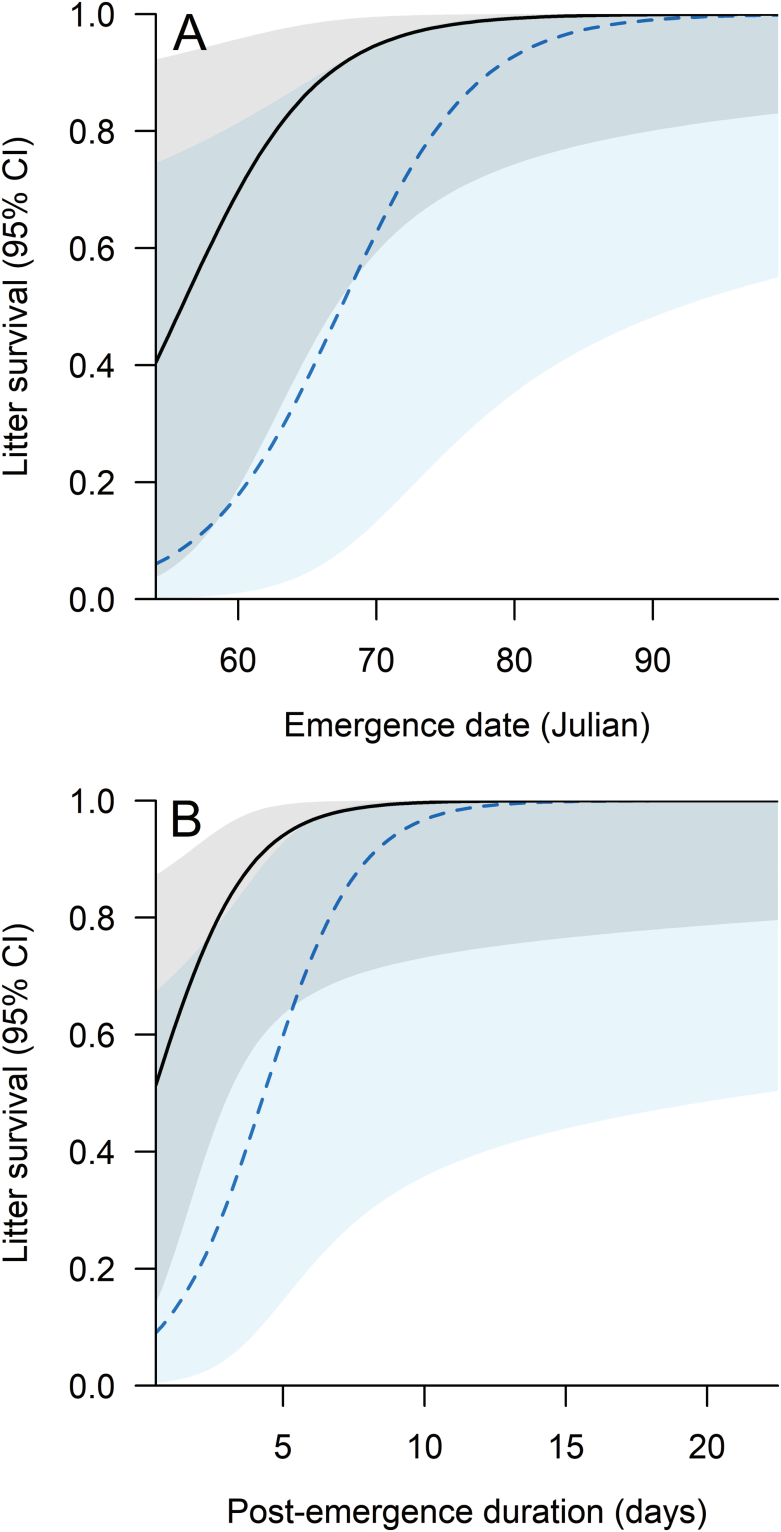
Expected influence of emergence date (A) and duration of the post-emergence denning period (B) on survival of 31 polar bear litters from dens on land (solid black line, gray CI) and sea ice (dashed blue line and CI) in the spring following denning. Other model terms were held constant at their mean values: duration of the survival interval = 25.5 days; post-emergence duration = 6.9 days (emergence date curve only); and emergence date = 74.9 (duration curve only).

For bears with emergence dates ≥27 February that were observed after den departure (*n* = 31), spring litter survival was influenced by emergence date, denning substrate (both of which were included in all candidate models), duration of the survival interval, and post-emergence duration (Table 2). Litter survival increased strongly with increasing emergence date and duration of the post-emergence period (Table 4; Fig. 4). Predicted survival rates increased from 0.5 to 0.9 when emergence date increased by approximately 10 days, with survival surpassing 0.9 when emergence date exceeded 8 March for dens on land and 19 March for dens on sea ice (other variables held at mean values; Fig. 4). Similarly, predicted survival rates increased from 0.5 to 0.9 when duration of the post-emergence period was increased by approximately 4 days, with survival surpassing 0.9 when duration of the post-emergence period exceeded 4 days for dens on land and 7 days for dens on sea ice (Fig. 4).

## Discussion

We expanded the temperature-based approach developed by Olson et al. (2017) and used by Rode et al. (2018b) to estimate dates of den entrance and emergence to successfully identify the phenology of the post-emergence denning period. This method, which should be applicable to other denning species, enabled us to estimate dates of emergence and departure for dens spanning large temporal and spatial extents, including those on sea ice where drift confounds the identification of denning from location data. Bears that were observed with cubs in the spring following denning stayed at the den site, on average, over 4 times longer than those that were observed without cubs (7.9 vs. 1.9 days; Table 3). After accounting for the influence of emergence date, denning substrate, and the duration of the survival interval (i.e. the time between den departure and observation), we found that duration of the post-emergence period was strongly associated with litter survival in the spring, when rates of cub mortality tend to be highest (Derocher and Stirling 1996). Specifically, we found that predicted litter survival rates increased from 0.5 to 0.9 when duration of the post-emergence period was increased by approximately 4 days. Assuming an emergence date of 16 March and a survival interval of 26 days, for example, litter survival surpassed 0.9 when post-emergence duration exceeded 4 days for dens on land and 7 days for dens on sea ice (Fig. 4). This increase in survival supports the possibility that there are selective advantages to remaining at the den site after emergence, presumably to allow cubs time to acclimate to ambient conditions and attain the strength necessary for traversing sea ice topography (Hansson and Thomassen 1983; Messier et al. 1994). Acclimation may be important for adult females also, but short post-emergence durations at dens with very early emergence dates—when viable cubs were less likely to be present—suggest that presence of cubs may govern how long bears remain at the den site (Fig. 2). Although duration of the post-emergence period appears to exert a strong influence on litter survival, it is possible that this relationship is confounded by other factors. For example, females in good body condition in the fall prior to denning are more likely to produce cubs that are larger at emergence and have higher survival rates than those of females in poor condition (Derocher and Stirling 1996; Robbins et al. 2012). Ostensibly, these females are also more likely to have energy reserves in the spring that allow them to remain at the den site longer post-emergence than bears in poor condition that may be forced to quickly depart to hunt. Future research incorporating factors known to influence cub survival (e.g. female age and previous reproductive success) could improve our understanding of what is likely a complex relationship between denning phenology and reproductive success.

Collecting data on polar bear den emergence and reproductive success is logistically challenging and it was often not possible to confirm presence or absence of cubs upon the emergence of denning females. Accordingly, our evaluation of the relationship between post-emergence duration and litter survival assumed that most failed pregnancies manifest earlier in the denning period (but see Ramsay and Andriashek 1986), and the dens we considered contained viable cubs at emergence. We attempted to limit the potential for including unproductive dens (i.e. those without viable cubs at emergence) by excluding those with emergence dates prior to that of the earliest known-productive den (27 February). Although viable cubs can emerge from dens earlier in February and potentially in January (Rode et al. 2018b), dens with January emergences are unlikely to be productive because cubs lack the ability to thermoregulate outside of the den until they reach a minimum of 60 to 70 days of age (Blix and Lentfer 1979; Kenny and Bickel 2005), and births typically occur in December or January (Lentfer and Hensel 1980; Messier et al. 1994). In our data set, post-emergence durations for dens with January emergences were typically very short (mean = 1.4 days) relative to those with emergence dates ≥27 February (mean = 8.8 days; Fig. 2), suggesting that bears typically do not remain at den sites long without viable cubs. Dens with February emergence dates prior to 27 February were characterized by durations (mean = 0.7 days) even shorter than those that emerged in January, which supports our approach of using 27 February as the threshold for analysis of litter survival (Fig. 2). Additionally, for denning bears that were observed later in the spring, all that emerged prior to 27 February were observed without cubs; in contrast, of those that emerged after the threshold but had short post-emergence durations typical of emergences prior to the threshold (≤1.4 days; *n* = 9), 78% were observed with cubs (Fig. 2). This difference in post-emergence duration points to the likely complexity of the relationship between litter survival and the post-emergence period. Fitness costs associated with short times at the den post-emergence could be offset by gains associated with later emergence dates (Fig. 4), and other traits linked to cub survival—such as female body condition—are likely to play a role in governing both denning phenology and success.

Although we believe it is unlikely that viable cubs were present at emergence at dens excluded from the analysis, we note that it is possible that cubs were lost prior to emergence at some dens that were included (i.e. those that emerged ≥27 February). It seems unlikely, however, that an adult female would remain in a den until March or April without viable cubs, especially considering the prolonged fast she had undergone by that time. This assumption is supported by observations of 25 dens that were monitored during the post-emergence period in northern Alaska, all of which emerged between 1 and 28 March and contained viable cubs (Smith et al. 2007, 2013; Robinson 2014). Additional information on the percent of female polar bears emerging from dens without cubs is limited. Derocher et al. (1992) estimated that 8% of pregnant females in western Hudson Bay aborted or did not implant in the 1980s, although it is unknown if those females may have emerged from dens early. Similarly, of females captured leaving the denning area in February and March 1982 to 1992, between 9% and 14% were without cubs (Derocher and Stirling 1995). In our study, 6 of 31 (19.4%) females who emerged from dens ≥27 February were observed later in the spring without cubs. Although our assumption that these losses occurred after den departure was informed by prior studies in Alaska (Smith et al. 2007, 2013; Robinson 2014) and further supported by the similarity in mean emergence date between dens that were known to be productive (17 March) and all dens that emerged ≥27 February (16 March), we note that the magnitude of the estimated effect of post-emergence duration on subsequent litter survival would be smaller had any of these dens been excluded from the analysis because they were known to have not produced viable cubs at emergence.

Our estimates of mean emergence are similar to dates reported for dens monitored by observers in Svalbard, Norway (17 to 18 March; Hansson and Thomassen 1983) and near Prudhoe Bay, Alaska (15 March; Smith et al. 2007, 2013), and to those estimated with the temperature-based approach used by Rode et al. (2018b) and calculated for analogous den-groups with the provided data (USGS 2018). Emergence estimates from Rode et al. (2018b) for all bears (3 March), those observed without cubs (21 February), and those observed with cubs (11 March) were earlier than our estimates by 4, 2, and 6 days, respectively (Table 3). These differences are likely the result of the inclusion of several bears with early estimated emergence dates in the Rode et al. (2018b) data set that did not meet our criteria for data-recording frequency necessary for separating emergence and departure dates or were estimated to have later emergences in our analysis. Similar to Rode et al. (2018b), we found that mean emergence date was later in the CS than in the SB, but the effect was smaller (5 days vs. 9 days) and statistical evidence for a difference was weak in our study (*P* = 0.11). Similarly, both studies found that mean emergence occurred later for dens on land than on sea ice, but the difference was smaller in our study (6 days vs. 9 days) and was not statistically significant (*P* = 0.25). These weaker effects were likely the result of differing objectives between the 2 studies. Whereas Rode et al. (2018b) investigated phenology of all dens, we focused on those that were likely productive and did not consider dens with emergence dates prior to 27 February in analyses.

Our estimate of mean den departure date (24 March) agreed with estimates from observer-based monitoring in Alaska (21 March; Smith et al. 2013) and collar sensor date from the Canadian Arctic archipelago (21 March; Messier et al. 1994). These departure estimates are later, however, than those reported from western Hudson Bay for 1998 to 1999 (6 March; Lunn et al. 2004) and 2011 to 2016 (1 March; Yee et al. 2017), which suggests that departure may vary geographically. Similar to den emergence, we found that den departure occurred 6 days later in the CS relative to the SB, and 7 days later on land relative to sea ice, but evidence for the latter difference was weak (*P* = 0.12). These differences may be somewhat confounded by dissimilarities in the distribution of dens between the 2 subpopulations. In the CS, Rode et al. (2015) estimated that 92% of dens occurred on land from 1986 to 1995 and 84% from 2008 to 2013 (88% in our sample), whereas in the SB, Olson et al. (2017) estimated that 34% of dens occurred on land from 1985 to 1995 and 55% from 2007 to 2013 (63% in our sample).

Bears varied greatly in how long they remained at the den site post-emergence, with duration lengths ranging from 0 to 32 days (Fig. 2). Our estimate of the mean duration of the post-emergence period (8.8 days) was similar to the collective estimate from 3 studies comprised of 25 dens that were monitored by observers in Alaska between 2002 and 2010 (8.0 ± 1.1 days; Smith et al. 2007, 2013; Robinson 2014) and for dens in Hudson Bay, Canada, for which emergence and departure were estimated with a combination of location, temperature, and activity sensor date (8.7 ± 1.8 days, *n* = 8; Lunn et al. 2004). Our estimate was substantially less, however, than observer-based estimates from areas of high-density denning on Herald Island, Russia (15.5 ± 2.6 days, *n* = 6; Ovsyanikov 1998) and Svalbard, Norway (14.0 ± 1.2 days, *n* = 25; Hansson and Thomassen 1983), and a collar sensor-derived estimate from the Canadian Arctic archipelago (13 ± 3 days, *n* = 35; Messier et al. 1994).

Predicted duration of the post-emergence period increased with emergence date to a peak in early March in the SB and late March in the CS before decreasing in both subpopulations (other model terms held at mean values; Fig. 3). The decrease in duration following the peak of each curve met our expectation that cubs that emerged later in the season might require less time at the den post-emergence because they were more likely to be larger at emergence than cubs that emerged earlier, and therefore would require less time to increase mass prior to departure. The cause of the increase in duration with increasing emergence date prior to late March for the CS is unclear. It is possible that bears that emerged early in the CS may have lost cubs prior to emergence or had energy reserves that had been depleted, necessitating a quick departure from the den to commence foraging.

Changes in sea ice habitat and snowfall patterns associated with global warming (Webster et al. 2014; Laidre et al. 2015) have the potential to affect behaviors of denning bears during the post-emergence period. Across the Arctic, freeze-up is occurring increasingly later in the year and breakup is occurring earlier, with the minimum extent of sea ice decreasing at a rate of 13% per decade (Meier et al. 2021). These reductions in sea ice are especially pronounced in the Beaufort and Chukchi seas (Stern and Laidre 2016), where polar bears are increasingly spending time on land in the summer and fall (Rode et al. 2015, 2022; Atwood et al. 2016; Olson et al. 2017). Increases in the time bears spend on land have been associated with increases in fasting and decreases in body condition, which can be especially detrimental to the reproductive success of denning females (Stirling et al. 1999; Rode et al. 2014; but see Mckinney et al. 2017). The positive relationship between post-emergence duration and litter survival could be influenced by a variety of other factors specific to each denning event, including cub size and development and female body condition (Garner et al. 1994). For example, because spring is a critical time for bears to replenish depleted energy reserves, females in poor condition may be forced to shorten the time they spend in dens and at the den site post-emergence in order to resume hunting, which is likely to impact cub survival. Identifying the ultimate factors that govern the decision of a bear on when to emerge and depart from dens would be especially useful in expanding our understanding of the link between den phenology and fitness.

The increase in denning on land in the SB has occurred concurrently with expansion of human activity within the range of polar bears (Rode et al. 2018a; Wilson and Durner 2020). Disturbance to dens may be especially impactful during the post-emergence period when dens are open and bears are often present on the surface where they may be exposed to stimuli directly, potentially resulting in abbreviated time at the den site. Larson et al. (2020), for example, noted that 3 dens in Alaska near sustained human activity were vacated 3 days after emergence, a period markedly shorter than the mean duration from this study (8.8 days). Because litter survival increases with increased time at the den site post-emergence, human actions that contribute to bears departing dens earlier than would have occurred under undisturbed conditions have the potential to impact cub production. This finding highlights the importance of mitigation actions that reduce exposure of denning bears to human activity post-emergence, such as continued use of buffers of restricted activity around dens. Additionally, the linkage of litter survival to emergence date and post-emergence duration could be incorporated into simulation exercises used to estimate the probability that proposed human activities would contribute to cub mortality, which is an important requirement for adherence to terms of the Marine Mammal Protection Act (Woodruff et al. 2022). Finally, developing methods that confirm the presence and number of cubs at emergence would be useful in expanding our understanding of the relationship between den phenology and cub survival.

## Supplementary data

Supplementary data are available at *Journal of Mammalogy* online.

**Supplementary Data SD1.**—Estimated dates of den emergence and departure calculated with temperature and location data and associated estimates of post-emergence duration for 70 bears collared in the Chukchi Sea and Southern Beaufort Sea subpopulations.

gyae010_suppl_Supplementary_Datas_SD1

## Data Availability

The data used in this study are available at: https://doi.org/10.5066/F7G73BTD (USGS and USFWS 2023).
