# Supplementary material for: The post-emergence period for denning Polar Bears: phenology and influence on cub survival
Source: J Mammal. 2024 Mar 2;105(3):490–501. doi: 10.1093/jmammal/gyae010 (PMC11130516; doi:10.1093/jmammal/gyae010)
Supplement: gyae010_suppl_Supplementary_Datas_SD1 [file gyae010_suppl_supplementary_datas_sd1.docx]

Supplementary Data SD1. Estimated dates (Julian) of den emergence (E) and departure (D) calculated with temperature and location data and associated estimates of post-emergence duration (Dur) for 70 bears collared in the Chukchi Sea (CS) and Southern Beaufort Sea (SB) subpopulations (Pop), along with denning substrate (Sub) and whether cubs were observed with the mother in the spring following denning (Cub; 1=cubs present, 0=absent). Minimum (min) and maximum (max) dates and durations are provided to account for uncertainty attributable to variability in recording intervals across sensors and GPS collars; midpoint estimates (mid) are dates and durations halfway between minimum and maximum estimates. If temperature-based estimates of departure for a den were outside of the range of location-based estimates or overlapped but were less precise (i.e., the span between minimum and maximum estimates was larger), we considered location-based estimates to be more accurate and used those dates for calculating post-emergence duration.

|  |  |  |  | Temperature | | | | | |  | Location | | |  |  |  |
| --- | --- | --- | --- | --- | --- | --- | --- | --- | --- | --- | --- | --- | --- | --- | --- | --- |
| Den | Pop | Sub | Cub | E_min_ | E_max_ | E_mid_ | D_min_ | D_max_ | D_mid_ |  | D_min_ | D_max_ | D_mid_ | Dur_min_ | Dur_max_ | Dur_mid_ |
| 1 | SB | NA | 0 | 11 | 12 | 11.5 | 11 | 12 | 11.5 |  | NA | NA | NA | 0 | 1 | 0.5 |
| 2 | CS | land | NA | 16 | 17 | 16.5 | 16 | 17 | 16.5 |  | 16 | 18 | 17 | 0 | 1 | 0.5 |
| 3 | CS | land | NA | 17 | 18 | 17.5 | 17 | 18 | 17.5 |  | NA | NA | NA | 0 | 1 | 0.5 |
| 4 | CS | land | NA | 20 | 21 | 20.5 | 28 | 29 | 28.5 |  | 29 | 29 | 29 | 8 | 9 | 8.5 |
| 5 | SB | NA | NA | 22 | 23 | 22.5 | 22 | 23 | 22.5 |  | NA | NA | NA | 0 | 1 | 0.5 |
| 6 | SB | land | 0 | 24 | 25 | 24.5 | 24 | 25 | 24.5 |  | NA | NA | NA | 0 | 1 | 0.5 |
| 7 | CS | land | NA | 25 | 26 | 25.5 | 25 | 26 | 25.5 |  | 22 | 24 | 23 | 0 | 0 | 0 |
| 8 | CS | ice | NA | 28 | 29 | 28.5 | 28 | 29 | 28.5 |  | NA | NA | NA | 0 | 1 | 0.5 |
| 9 | SB | ice | NA | 29 | 31 | 30 | 29 | 31 | 30 |  | NA | NA | NA | 0 | 2 | 1 |
| 10 | SB | land | 0 | 29 | 34 | 31.5 | 29 | 34 | 31.5 |  | 29 | 34 | 31.5 | 0 | 5 | 2.5 |
| 11 | CS | land | NA | 28 | 40 | 34 | 28 | 40 | 34 |  | 28 | 28 | 28 | 0 | 0 | 0 |
| 12 | SB | land | 0 | 46 | 47 | 46.5 | 46 | 47 | 46.5 |  | 31 | 46 | 38.5 | 0 | 0 | 0 |
| 13 | CS | land | NA | 48 | 49 | 48.5 | 48 | 49 | 48.5 |  | 46 | 52 | 49 | 0 | 1 | 0.5 |
| 14 | SB | ice | 0 | 51 | 52 | 51.5 | 51 | 52 | 51.5 |  | NA | NA | NA | 0 | 1 | 0.5 |
| 15 | SB | land | 0 | 51 | 58 | 54.5 | 51 | 58 | 54.5 |  | 30 | 61 | 45.5 | 0 | 7 | 3.5 |
| 16 | SB | ice | NA | 52 | 61 | 56.5 | 73 | 76 | 74.5 |  | NA | NA | NA | 12 | 24 | 18 |
| 17 | SB | land | 1 | 56 | 58 | 57 | 68 | 70 | 69 |  | 61 | 70 | 65.5 | 10 | 14 | 12 |
| 18 | SB | ice | NA | 55 | 59 | 57 | 77 | 78 | 77.5 |  | NA | NA | NA | 18 | 23 | 20.5 |
| 19 | SB | land | NA | 56 | 59 | 57.5 | 56 | 59 | 57.5 |  | 56 | 62 | 59 | 0 | 3 | 1.5 |
| 20 | SB | land | 1 | 58 | 61 | 59.5 | 65 | 66 | 65.5 |  | 67 | 71 | 69 | 6 | 13 | 9.5 |
| 21 | CS | land | NA | 61 | 62 | 61.5 | 65 | 66 | 65.5 |  | 66 | 68 | 67 | 4 | 5 | 4.5 |
| 22 | SB | land | 1 | 61 | 62 | 61.5 | 71 | 72 | 71.5 |  | 72 | 72 | 72 | 10 | 11 | 10.5 |
| 23 | SB | land | NA | 61 | 62 | 61.5 | 89 | 90 | 89.5 |  | 93 | 93 | 93 | 31 | 32 | 31.5 |
| 24 | SB | land | 1 | 62 | 63 | 62.5 | 62 | 63 | 62.5 |  | 61 | 64 | 62.5 | 0 | 2 | 1 |
| 25 | SB | ice | NA | 60 | 66 | 63 | 69 | 72 | 70.5 |  | NA | NA | NA | 3 | 12 | 7.5 |
| 26 | SB | land | 1 | 63 | 65 | 64 | 77 | 78 | 77.5 |  | 76 | 76 | 76 | 11 | 13 | 12 |
| 27 | SB | ice | 0 | 64 | 65 | 64.5 | 64 | 65 | 64.5 |  | NA | NA | NA | 0 | 1 | 0.5 |
| 28 | SB | land | 1 | 66 | 67 | 66.5 | 79 | 80 | 79.5 |  | 79 | 80 | 79.5 | 12 | 14 | 13 |
| 29 | SB | ice | 1 | 67 | 69 | 68 | 76 | 78 | 77 |  | NA | NA | NA | 7 | 11 | 9 |
| 30 | SB | ice | 1 | 67 | 70 | 68.5 | 70 | 73 | 71.5 |  | NA | NA | NA | 0 | 6 | 3 |
| 31 | SB | land | 1 | 68 | 69 | 68.5 | 85 | 86 | 85.5 |  | 87 | 88 | 87.5 | 18 | 20 | 19 |
| 32 | SB | land | 0 | 68 | 70 | 69 | 76 | 77 | 76.5 |  | 75 | 80 | 77.5 | 6 | 9 | 7.5 |
| 33 | CS | ice | 1 | 69 | 70 | 69.5 | 75 | 76 | 75.5 |  | NA | NA | NA | 5 | 7 | 6 |
| 34 | CS | ice | NA | 70 | 71 | 70.5 | 81 | 82 | 81.5 |  | NA | NA | NA | 10 | 12 | 11 |
| 35 | CS | land | NA | 70 | 71 | 70.5 | 75 | 85 | 80 |  | 76 | 85 | 80.5 | 5 | 15 | 10 |
| 36 | CS | land | NA | 71 | 72 | 71.5 | 73 | 74 | 73.5 |  | 74 | 74 | 74 | 2 | 3 | 2.5 |
| 37 | SB | ice | NA | 71 | 72 | 71.5 | 74 | 75 | 74.5 |  | NA | NA | NA | 2 | 4 | 3 |
| 38 | SB | land | 1 | 72 | 73 | 72.5 | 87 | 88 | 87.5 |  | 93 | 97 | 95 | 20 | 25 | 22.5 |
| 39 | CS | land | NA | 73 | 74 | 73.5 | 73 | 74 | 73.5 |  | 73 | 76 | 74.5 | 0 | 1 | 0.5 |
| 40 | CS | land | NA | 73 | 74 | 73.5 | 94 | 95 | 94.5 |  | 94 | 98 | 96 | 20 | 22 | 21 |
| 41 | SB | ice | NA | 73 | 74 | 73.5 | 82 | 83 | 82.5 |  | NA | NA | NA | 8 | 10 | 9 |
| 42 | CS | land | NA | 70 | 78 | 74 | 79 | 80 | 79.5 |  | 81 | 81 | 81 | 3 | 11 | 7 |
| 43 | SB | ice | 1 | 74 | 75 | 74.5 | 101 | 102 | 101.5 |  | 96 | 96 | 96 | 21 | 22 | 21.5 |
| 44 | SB | land | 1 | 74 | 77 | 75.5 | 74 | 77 | 75.5 |  | 68 | 77 | 72.5 | 0 | 3 | 1.5 |
| 45 | SB | ice | 0 | 74 | 77 | 75.5 | 74 | 77 | 75.5 |  | NA | NA | NA | 0 | 3 | 1.5 |
| 46 | SB | ice | NA | 74 | 77 | 75.5 | 77 | 80 | 78.5 |  | NA | NA | NA | 0 | 6 | 3 |
| 47 | SB | land | 1 | 75 | 76 | 75.5 | 79 | 80 | 79.5 |  | 77 | 77 | 77 | 1 | 2 | 1.5 |
| 48 | SB | land | 1 | 77 | 78 | 77.5 | 77 | 78 | 77.5 |  | 67 | 79 | 73 | 0 | 1 | 0.5 |
| 49 | CS | land | NA | 78 | 79 | 78.5 | 101 | 102 | 101.5 |  | 102 | 102 | 102 | 23 | 24 | 23.5 |
| 50 | SB | land | 1 | 80 | 81 | 80.5 | 92 | 93 | 92.5 |  | 89 | 94 | 91.5 | 11 | 13 | 12 |
| 51 | SB | ice | NA | 80 | 81 | 80.5 | 89 | 90 | 89.5 |  | NA | NA | NA | 8 | 10 | 9 |
| 52 | CS | land | NA | 81 | 82 | 81.5 | 99 | 100 | 99.5 |  | 98 | 101 | 99.5 | 17 | 19 | 18 |
| 53 | SB | ice | 0 | 79 | 85 | 82 | 79 | 85 | 82 |  | NA | NA | NA | 0 | 6 | 3 |
| 54 | SB | land | 0 | 82 | 83 | 82.5 | 82 | 83 | 82.5 |  | 81 | 83 | 82 | 0 | 1 | 0.5 |
| 55 | SB | land | 1 | 82 | 83 | 82.5 | 90 | 91 | 90.5 |  | 97 | 97 | 97 | 14 | 15 | 14.5 |
| 56 | SB | land | 1 | 82 | 83 | 82.5 | 82 | 83 | 82.5 |  | 82 | 85 | 83.5 | 0 | 1 | 0.5 |
| 57 | CS | land | NA | 83 | 84 | 83.5 | 101 | 102 | 101.5 |  | 102 | 104 | 103 | 18 | 19 | 18.5 |
| 58 | CS | land | NA | 83 | 84 | 83.5 | 96 | 97 | 96.5 |  | 97 | 97 | 97 | 13 | 14 | 13.5 |
| 59 | SB | land | 1 | 83 | 84 | 83.5 | 97 | 98 | 97.5 |  | 100 | 100 | 100 | 16 | 17 | 16.5 |
| 60 | SB | ice | NA | 82 | 87 | 84.5 | 82 | 87 | 84.5 |  | NA | NA | NA | 0 | 5 | 2.5 |
| 61 | SB | land | 1 | 83 | 86 | 84.5 | 83 | 86 | 84.5 |  | 86 | 89 | 87.5 | 0 | 3 | 1.5 |
| 62 | CS | land | NA | 85 | 86 | 85.5 | 99 | 100 | 99.5 |  | 99 | 101 | 100 | 13 | 15 | 14 |
| 63 | SB | land | 1 | 85 | 86 | 85.5 | 85 | 86 | 85.5 |  | 84 | 87 | 85.5 | 0 | 1 | 0.5 |
| 64 | SB | land | 1 | 85 | 86 | 85.5 | 85 | 86 | 85.5 |  | NA | NA | NA | 0 | 1 | 0.5 |
| 65 | CS | land | NA | 87 | 88 | 87.5 | 94 | 95 | 94.5 |  | 97 | 97 | 97 | 9 | 10 | 9.5 |
| 66 | CS | land | NA | 88 | 89 | 88.5 | 102 | 103 | 102.5 |  | NA | NA | NA | 13 | 15 | 14 |
| 67 | SB | ice | 1 | 92 | 94 | 93 | 98 | 100 | 99 |  | NA | NA | NA | 4 | 8 | 6 |
| 68 | CS | land | NA | 94 | 95 | 94.5 | 94 | 95 | 94.5 |  | 96 | 98 | 97 | 1 | 4 | 2.5 |
| 69 | SB | land | 1 | 92 | 98 | 95 | 92 | 98 | 95 |  | 90 | 99 | 94.5 | 0 | 6 | 3 |
| 70 | SB | land | 1 | 98 | 100 | 99 | 98 | 100 | 99 |  | 70 | 100 | 85 | 0 | 2 | 1 |
